# Long‐Term Side Effects of Breast Cancer Treatments: A Systematic Review

**DOI:** 10.1155/tbj/6873605

**Published:** 2026-06-25

**Authors:** Ebrahim Babaee, Mahdi Soheyli, Nahid Nafissi

**Affiliations:** ^1^ Preventive Medicine and Public Health Research Center, Psychosocial Health Research Institute, Community and Family Medicine Department, School of Medicine, Iran University of Medical Sciences, Tehran, Iran, iums.ac.ir; ^2^ Preventive Medicine and Public Health Research Center, Psychosocial Health Research Institute, Community and Family Medicine Department, School of Medicine, Iran University of Medical Sciences, Tehran, Iran, iums.ac.ir; ^3^ Breast Health & Cancer Research Center, Iran University of Medical Sciences (IUMS), Tehran, Iran, iums.ac.ir

**Keywords:** breast cancer, chemotherapy, endocrine therapy, long-term side effects, quality of life, radiotherapy, survivorship

## Abstract

**Background:**

Breast cancer treatments, including chemotherapy, radiotherapy, endocrine therapy, targeted therapy, and surgical interventions, have significantly improved survival rates. However, these treatments are associated with long‐term side effects that can impact the quality of life of survivors. Understanding these adverse effects is crucial for optimizing survivorship care.

**Methods:**

This systematic review was conducted following PRISMA guidelines to assess the long‐term side effects of breast cancer treatments. A comprehensive literature search was performed for studies published between 2005 and 2024. Studies examining long‐term (≥ 12 months posttreatment) adverse effects in breast cancer survivors were included, with data extraction and risk of bias assessments conducted by independent reviewers.

**Results:**

The review identified a broad spectrum of long‐term side effects, including cardiovascular complications, cognitive impairment, persistent fatigue, lymphedema, menopausal symptoms, and psychological distress. Chemotherapy was frequently associated with peripheral neuropathy and cognitive decline, while radiotherapy increased the risk of fibrosis, secondary malignancies, and ischemic heart disease. Endocrine therapy contributed to osteoporosis, joint pain, and metabolic disturbances, whereas HER2‐targeted therapies were linked to cardiotoxicity. In addition, surgical interventions, particularly axillary lymph node dissection, were a primary cause of lymphedema. Psychological distress, including anxiety, depression, and posttraumatic stress disorder, was also prevalent among survivors.

**Conclusion:**

The long‐term side effects of breast cancer treatments highlight the need for comprehensive survivorship care, including routine monitoring, personalized rehabilitation programs, lifestyle modifications, and psychosocial support. Future research should focus on identifying risk factors, developing targeted interventions, and optimizing treatment strategies to minimize adverse effects and improve the quality of life for breast cancer survivors.

## 1. Introduction

Breast cancer is the most commonly diagnosed malignancy among women worldwide, accounting for approximately 2.3 million new cases annually [1]. Advances in early detection, surgical techniques, systemic therapies, and radiotherapy have significantly improved survival rates over the past few decades [2]. As a result, the number of long‐term breast cancer survivors continues to grow, emphasizing the importance of not only achieving remission but also ensuring long‐term quality of life. Despite these advancements, breast cancer treatments are associated with a spectrum of long‐term adverse effects that can persist for years after treatment completion, affecting multiple aspects of physical and psychological well‐being [3, 4]. Understanding these side effects is crucial for developing effective survivorship care strategies and improving posttreatment patient outcomes.

Long‐term complications vary based on the treatment modality received. Chemotherapy‐induced toxicity is a major concern, with anthracyclines and taxanes frequently associated with persistent peripheral neuropathy, cognitive dysfunction (“chemo brain”), and cardiotoxicity [5, 6]. Radiotherapy, while highly effective in reducing recurrence, can lead to fibrosis, secondary malignancies, and radiation‐induced heart disease, particularly in patients receiving treatment for left‐sided breast cancer [7]. Endocrine therapy, commonly prescribed for hormone receptor–positive breast cancer, is linked to osteoporosis, joint pain, and metabolic disturbances, while targeted therapies such as trastuzumab (HER2 inhibitors) can result in cardiotoxic effects [6, 8]. In addition, surgical procedures, particularly axillary lymph node dissection and mastectomy, contribute to physical limitations, chronic pain, and lymphedema, which can impact mobility and daily activities [9].

Beyond physical complications, long‐term breast cancer survivors often experience significant psychological and cognitive challenges. Anxiety, depression, posttraumatic stress, and persistent cancer‐related fatigue are prevalent among survivors, affecting overall well‐being and functional capacity [10, 11]. Furthermore, treatment‐related hormonal changes can induce menopausal symptoms, sexual dysfunction, and infertility, posing additional emotional and social burdens [12]. The interplay between these physical and psychological sequelae highlights the need for a comprehensive, multidisciplinary approach to survivorship care, encompassing medical follow‐ups, rehabilitation programs, and psychosocial support.

Given the complexity and variability of these long‐term effects, a systematic synthesis of current evidence is essential to guide clinical decision‐making and survivorship care planning. This review aims to evaluate and summarize the long‐term adverse effects associated with various breast cancer treatment modalities. By analyzing existing literature, this study seeks to provide insights into the prevalence, risk factors, and management strategies for these complications. The findings will contribute to improving patient outcomes by promoting early detection, preventive measures, and personalized treatment approaches tailored to survivorship needs.

## 2. Study Design

This study is a systematic review conducted in 2025 according to the guidelines outlined in the PRISMA framework. The review aims to review current evidence on the long‐term side effects associated with breast cancer treatments, including chemotherapy, radiotherapy, endocrine therapy, targeted therapy, and surgical interventions.

### 2.1. Eligibility Criteria

In this study, studies were considered eligible for inclusion if they met the following criteria:

• Published in peer‐reviewed journals between 2005 and 2024.
• Studies investigating long‐term (≥ 12 months posttreatment) adverse effects in breast cancer survivors.
• Research focusing on chemotherapy, radiotherapy, endocrine therapy, targeted therapy, or surgical interventions.
• Observational studies, randomized controlled trials (RCTs), cohort studies, case‐control studies, and systematic reviews.
• Studies written in English.

### 2.2. Exclusion Criteria

Studies were excluded if they met any of the following criteria:

• Research limited to preclinical (animal) studies, editorials, conference abstracts, or case reports.
• Studies that primarily focus on breast cancer recurrence, treatment efficacy, or survival rates without discussing long‐term side effects.
• Articles not available in full‐text format.

With the study objectives specified, the study components are presented in Table 1 using the population, intervention, comparator, and outcome (PICO) framework.

**TABLE 1:** PICO framework for describing the components.

Component	Description
Population (P)	Breast cancer survivors
Intervention (I)	Cancer treatments (chemotherapy, radiotherapy, endocrine therapy, and surgery)
Comparison (C)	No treatment or alternative treatments
Outcome (O)	Long‐term side effects (cardiovascular complications, cognitive impairment, fatigue, lymphedema, menopausal symptoms, and psychological distress)

### 2.3. Search Strategy

A comprehensive literature search was conducted in PubMed, Scopus, Web of Science, and Cochrane Library as electronic databases and Google Scholar for additional gray literature as a search engine.

The search strategy included a combination of Medical Subject Headings (MeSH) terms and free‐text keywords. The following terms were used as query search:

(“Breast Cancer” OR “Breast Neoplasms”) AND (“Long‐term Side Effects” OR “Late Effects” OR “Chronic Toxicity”) AND (“Chemotherapy” OR “Radiotherapy” OR “Endocrine Therapy” OR “Targeted Therapy” OR “Surgery”) AND (“Cardiotoxicity” OR “Peripheral Neuropathy” OR “Lymphedema” OR “Cognitive Dysfunction” OR “Osteoporosis” OR “Menopause” OR “Fatigue” OR “Mental Health Disorders” OR “Quality of Life”).

**FIGURE 1:**
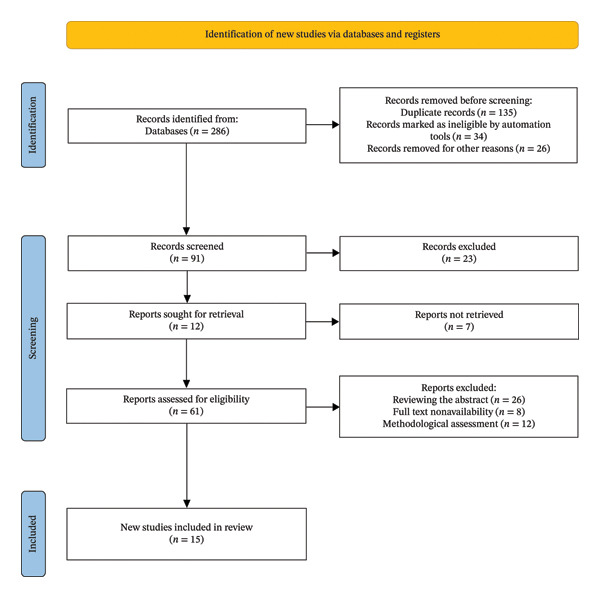
The PRISMA flowchart.

Also, reference lists of relevant articles and systematic reviews were screened for additional eligible studies. After searching the databases, 286 articles were selected for initial evaluation. After screening the selected articles by reviewing the abstract, full text, methodological assessment, and applying duplication process, 15 articles were finally selected for evaluation and reporting of the results. The process of selecting and evaluating articles using the PRISMA flowchart is shown in Figure 1.

Although the search covered a broad time frame (2005–2024), the final number of included studies was limited due to strict inclusion criteria focusing on long‐term (≥ 12 months) outcomes and the exclusion of studies lacking comprehensive follow‐up data.

### 2.4. Study Selection and Data Extraction

After removing duplicate studies, two independent reviewers (blinded to each other’s selection) screened the titles and abstracts for relevance. Full‐text articles of selected studies were reviewed, and any disagreements were resolved through discussion or by a third reviewer. A standardized data extraction form was used to collect information, including study characteristics (author, year, study design, and sample size), population details (age, breast cancer stage, and treatment received), long‐term side effects reported (e.g., cardiotoxicity, neuropathy, lymphedema, osteoporosis, and cognitive impairment), follow‐up duration, key findings, and conclusions.

### 2.5. Quality Assessment and Risk of Bias

The methodological quality of included studies was assessed using the ROBINS‐I tool, and finally, studies were classified as low, moderate, or high risk of bias (Table 2). Two independent reviewers (blinded to each other’s selection) screened the articles in terms of risk of bias appraisal, and any disagreements were resolved through discussion or by a third reviewer.

**TABLE 2:** Summary of risk of bias assessment using ROBINS‐I tool.

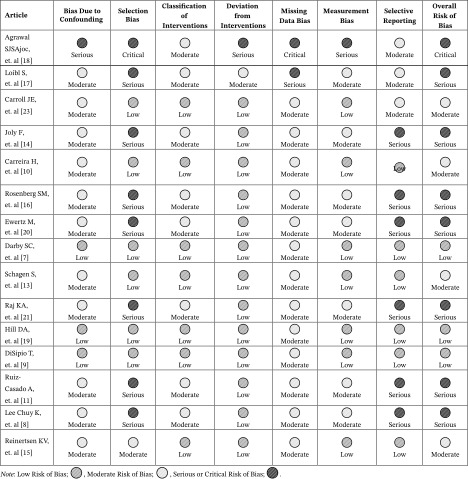

### 2.6. Ethical Considerations

As this study is a systematic review of publicly available literature, no ethical approval was required. However, the review adhered to ethical guidelines by ensuring transparency and avoiding any publication and reporting biases.

## 3. Results

Breast cancer treatments, including surgery, radiotherapy, chemotherapy, and endocrine therapy, have been associated with a range of long‐term side effects that significantly impact survivors’ quality of life. The systematic review identified multiple domains of late effects, including cardiovascular complications, cognitive impairment, fatigue, lymphedema, menopausal symptoms, and psychological distress. These side effects vary in severity and duration, often persisting for years after treatment completion.

### 3.1. Cardiovascular Complications

Radiotherapy and certain systemic therapies have been linked to increased cardiovascular risks among breast cancer survivors. Darby et al. (2013) reported that women receiving radiotherapy for breast cancer have a significantly increased risk of ischemic heart disease, with the risk rising proportionally to the radiation dose received by the heart. The study highlighted that even small doses of radiation could elevate the risk, making cardiac monitoring crucial [7]. In addition, Lee Chuy and Yu (2019) discussed the cardiotoxic effects of contemporary breast cancer treatments, particularly anthracyclines and trastuzumab. These agents have been associated with an increased risk of heart failure and long‐term cardiac dysfunction, necessitating regular cardiovascular assessment and early intervention strategies [8].

### 3.2. Cognitive Impairment

Cognitive dysfunction, often referred to as “chemo brain,” is a persistent issue for many breast cancer survivors. Schagen et al. (2002) demonstrated that adjuvant chemotherapy has long‐term detrimental effects on cognitive function, including memory deficits, reduced processing speed, and attention difficulties [19]. Carreira et al. further linked cognitive decline to biological markers of aging, suggesting that chemotherapy and endocrine therapy may accelerate neurobiological aging processes. These cognitive impairments can impact daily functioning, employment, and overall quality of life, highlighting the need for cognitive rehabilitation and supportive interventions [10].

### 3.3. Cancer‐Related Fatigue

Fatigue is one of the most commonly reported long‐term side effects in breast cancer survivors. Reinertsen et al. and Joly et al. found that cancer‐related fatigue persists long after treatment completion, affecting survivors’ ability to perform daily activities, maintain employment, and engage in social interactions [16, 22]. Ruiz‐Casado et al. reinforced these findings, indicating that fatigue is a multifactorial issue influenced by both physiological changes due to treatment and psychosocial factors such as anxiety and depression. Strategies for managing fatigue include physical activity, cognitive‐behavioral therapy, and structured rehabilitation programs [11].

### 3.4. Lymphedema

Lymphedema, a chronic swelling of the arm due to lymphatic system damage, remains a prevalent concern for breast cancer survivors, particularly those who undergo axillary lymph node dissection or radiation therapy. DiSipio et al. conducted a systematic review and meta‐analysis, revealing that unilateral arm lymphedema affects approximately 21% of patients posttreatment. Lymphedema can cause physical discomfort, restricted movement, and an increased risk of infections such as cellulitis. Early detection and management, including physical therapy, compression garments, and lifestyle modifications, are essential for improving outcomes [9].

### 3.5. Menopausal Symptoms

Many breast cancer treatments, especially chemotherapy and endocrine therapy, induce premature menopause, significantly impacting the quality of life. Rosenberg and Ahjjotd noted that young breast cancer survivors often experience severe menopausal symptoms such as hot flashes, night sweats, vaginal dryness, decreased libido, and osteoporosis [17]. Loibl et al. emphasized the challenges in managing these symptoms, as hormone replacement therapy is often contraindicated due to concerns about cancer recurrence. Alternative management strategies, including nonhormonal medications, lifestyle modifications, and psychological support, are recommended to alleviate menopausal symptoms [14].

### 3.6. Psychological Distress

Breast cancer survivorship is also associated with increased risks of mental health disorders. Carreira et al. reported a higher incidence of anxiety, depression, and posttraumatic stress disorder among breast cancer survivors compared to the general population [10]. Agrawal highlighted the persistent emotional burden and social difficulties faced by survivors due to physical changes, fear of recurrence, and the long‐term impact of treatment‐related side effects. Psychological support, including counseling and peer support programs, plays a crucial role in improving mental health outcomes for survivors [13].

### 3.7. Other Long‐Term Effects

Additional complications, such as osteoporosis, chronic pain, and neuropathy, have been reported in the literature. Hill et al. noted a higher prevalence of osteoporosis in patients receiving endocrine therapy, particularly aromatase inhibitors, which lead to reduced bone density and increased fracture risk [21]. Ewertz and Jensen identified persistent pain and neuropathy as significant concerns posttreatment, often linked to chemotherapy‐induced nerve damage [18]. Raj et al. discussed the late effects of breast radiotherapy in young women, including fibrosis, skin changes, and an increased risk of secondary malignancies [20].

Overall, the findings from this systematic review reveal significant variations in long‐term side effects based on patient age and treatment type. Middle‐aged to older survivors, particularly those over 50, have a higher incidence of cardiovascular complications, with studies showing that radiotherapy increases the risk of ischemic heart disease by approximately 7.4% per gray of radiation delivered to the heart [7]. Fatigue is also prevalent, affecting up to 38% of long‐term survivors [22]. In addition, endocrine therapy, particularly aromatase inhibitors, is associated with a 54% increased risk of osteoporosis and fractures in postmenopausal women [21]. Conversely, younger survivors under 45 are disproportionately affected by premature menopause, with nearly 80% experiencing vasomotor symptoms such as hot flashes and night sweats [17]. Psychological distress is a pervasive issue across all age groups, with anxiety and depression rates being 24% higher in breast cancer survivors than in the general population [10].

The type of anticancer treatment strongly correlates with specific long‐term effects. Chemotherapy, particularly anthracyclines and taxanes, is a major contributor to cognitive impairment, with 30% of survivors reporting persistent cognitive decline, including memory deficits and decreased processing speed [19]. Trastuzumab, commonly used for HER2‐positive breast cancer, increases the risk of heart failure by 4%–7% [8]. Surgical interventions, such as axillary lymph node dissection, are closely linked to lymphedema, with an estimated incidence of 21% among affected patients [9]. Radiation therapy, while effective in reducing recurrence rates, has been associated with chronic pain, skin fibrosis, and secondary malignancies, particularly in younger women receiving high‐dose radiation [20]. Understanding these treatment‐specific risks is crucial for developing targeted survivorship care plans and improving long‐term outcomes for breast cancer survivors. The final summary of the detailed results of the study review is reported in Table 3.

**TABLE 3:** Summary of results of the reviewed studies.

Long‐term side effect	Key findings	Patient type	Age group	Country of study	Type of anticancer treatment performed	Comparison group	Time interval for occurrence	References
Cardiovascular complications	Radiotherapy and systemic therapies increase the risk of heart disease and heart failure. Monitoring is crucial	Breast cancer survivors	Middle‐aged to older adults	Multicountry (UK, USA, and Europe)	Radiotherapy, chemotherapy (anthracyclines, and trastuzumab)	Women without breast cancer or nonradiated breast cancer patients	5–20 years posttreatment	[7, 8]
Cognitive impairment	Chemotherapy and endocrine therapy contribute to cognitive decline, including memory deficits and slower processing speed	Breast cancer survivors	Middle‐aged women	Netherlands and USA	Chemotherapy and endocrine therapy	Women without chemotherapy treatment	6 months to 10 years posttreatment	[15, 19]
Cancer‐related fatigue	Persistent fatigue affects daily life, work, and social interactions. Influenced by physiological and psychosocial factors	Breast cancer survivors	Middle‐aged to older adults	Norway, France, and Spain	Chemotherapy and radiotherapy	General population without cancer	Months to years posttreatment	[11, 16, 22]
Lymphedema	Affects ∼21% of patients posttreatment, causing swelling, discomfort, and infection risk. Managed with therapy and compression	Breast cancer survivors	Adult women	Australia	Surgery (axillary lymph node dissection) and radiotherapy	Breast cancer patients without lymph node dissection	Within the first 3 years posttreatment, can persist indefinitely	[9]
Menopausal symptoms	Chemotherapy and endocrine therapy can cause severe menopause symptoms. HRT is often contraindicated	Young breast cancer survivors	Young to middle‐aged adults	USA	Chemotherapy and endocrine therapy	Women without cancer or those not on endocrine therapy	Immediate to long‐term posttreatment	[14, 17]
Psychological distress	Increased risk of anxiety, depression, and PTSD due to fear of recurrence and treatment effects. Support is essential	Breast cancer survivors	Adult women	UK and India	Various (chemotherapy, radiotherapy, and surgery)	General population or women with noncancer chronic illnesses	Ongoing, can persist for years	[10, 13]
Peripheral neuropathy	Persistent neuropathy and chronic pain are common after chemotherapy, affecting daily functioning and quality of life	Breast cancer survivors	Middle‐aged to older adults	Denmark	Chemotherapy	Women without chemotherapy exposure	Months to years posttreatment, may persist long‐term	[18]
Other long‐term effects	Includes osteoporosis, fibrosis, skin changes, and secondary malignancies	Breast cancer survivors	Middle‐aged to older adults	USA and India	Endocrine therapy, chemotherapy, and radiotherapy	Women without cancer or nontreated survivors	Varies by condition (years posttreatment for osteoporosis, neuropathy, and secondary malignancies)	[20, 21]

## 4. Discussion

The findings from this systematic review highlight the substantial burden of long‐term side effects associated with breast cancer treatments. As survival rates improve, understanding these persistent complications is essential for optimizing survivorship care and enhancing quality of life. The reviewed studies underscore that the nature and severity of long‐term effects vary based on treatment modality, patient demographics, and individual susceptibility.

One of the most concerning long‐term adverse effects of breast cancer treatments is cardiovascular toxicity, particularly in patients exposed to radiotherapy and anthracycline‐based chemotherapy. Studies by Darby et al. and Lee Chuy and Yu indicate that even low doses of radiation can increase the risk of ischemic heart disease, while anthracyclines and trastuzumab contribute to long‐term cardiac dysfunction [7, 8]. Recent studies suggest that the use of cardioprotective agents such as beta‐blockers and ACE inhibitors may help mitigate these risks [23]. Given these risks, routine cardiovascular monitoring and the development of cardioprotective strategies are crucial components of survivorship care.

Cognitive dysfunction, commonly referred to as “chemo brain,” persists in a significant subset of breast cancer survivors. Schagen et al. and Carroll et al. provided compelling evidence that chemotherapy and endocrine therapy accelerate neurobiological aging, leading to memory impairment, reduced processing speed, and attentional difficulties [15, 19]. These cognitive deficits have profound implications for daily functioning and employment, warranting further research into rehabilitative strategies, such as cognitive training and pharmacological interventions [24].

Fatigue remains one of the most commonly reported and debilitating long‐term side effects. Studies by Reinertsen et al. and Joly et al. suggest that this fatigue is multifactorial, influenced by both biological changes from treatment and psychosocial factors such as anxiety and depression [16, 22]. Physical activity, cognitive‐behavioral therapy, and structured rehabilitation programs have shown promise in mitigating this issue, emphasizing the need for individualized management approaches [11]. Emerging evidence suggests that mindfulness‐based interventions and yoga may provide additional benefits for managing persistent fatigue [25].

Lymphedema, often resulting from axillary lymph node dissection or radiotherapy, affects approximately 21% of breast cancer survivors, as demonstrated in the meta‐analysis by DiSipio et al. [9]. This chronic condition not only impacts physical function but also contributes to psychological distress. Early detection through regular monitoring and interventions such as compression therapy, manual lymphatic drainage, and exercise can significantly improve patient outcomes [26, 27].

Premature menopause induced by chemotherapy and endocrine therapy significantly affects young breast cancer survivors. The studies by Rosenberg and Partridge and Loibl et al. report that nearly 80% of younger survivors experience vasomotor symptoms such as hot flashes and night sweats, along with osteoporosis and sexual dysfunction [14, 17]. Given the contraindications for hormone replacement therapy in these patients, nonhormonal alternatives, lifestyle modifications, and psychological support should be prioritized [28, 29].

The psychological impact of breast cancer treatments is profound, with increased risks of anxiety, depression, and posttraumatic stress disorder. Carreira et al. and Agrawal and Mehnert highlight the long‐term emotional burden associated with treatment‐induced physical changes and fear of recurrence [10, 13, 30]. Survivorship care plans must integrate mental health support, including counseling, peer support groups, and mindfulness‐based interventions to improve long‐term psychological well‐being [31].

Additional complications such as osteoporosis, chronic pain, and neuropathy further complicate the posttreatment landscape. Endocrine therapy, particularly aromatase inhibitors, is associated with a 54% increased risk of osteoporosis and fractures [21]. Chemotherapy‐induced peripheral neuropathy, as discussed by Ewertz and Jensen, significantly affects daily functioning and pain management [18]. Furthermore, Raj et al. noted that radiation therapy increases the risk of skin fibrosis and secondary malignancies in younger women [20]. These findings emphasize the need for long‐term monitoring and multidisciplinary interventions to mitigate these risks.

The comprehensive assessment of long‐term side effects underscores the need for a personalized approach to survivorship care. Tailored follow‐up programs, early intervention strategies, and integrative care models involving oncologists, cardiologists, psychologists, and rehabilitation specialists can improve patient outcomes. Future research should focus on identifying biomarkers that predict long‐term toxicity, developing novel therapeutic strategies with fewer adverse effects, and implementing preventive measures to minimize treatment‐related morbidity.

This systematic review brings together current evidence on the long‐term side effects of breast cancer treatments, offering a broad perspective on their prevalence, clinical impact, and implications for survivorship care. By examining multiple treatment modalities and incorporating a multidisciplinary viewpoint, the findings highlight the importance of integrated, patient‐centered approaches to long‐term management. The inclusion of a wide range of studies also allows for practical insights that may inform both clinical practice and health policy.

At the same time, several limitations should be acknowledged. The heterogeneity among the included studies, along with the presence of moderate to high risk of bias in some—particularly those by Agrawal et al. and Loibl et al—may affect the strength of certain conclusions. These studies may be influenced by confounding factors, selection bias, and incomplete reporting, and therefore, findings related to outcomes such as psychological distress and menopausal symptoms should be interpreted with some caution. In contrast, greater confidence can be placed in results supported by studies with a lower risk of bias.

Finally, most of the available evidence is derived from Western populations, which may limit how well these findings apply to other settings. Differences in genetics, healthcare access, and survivorship care resources could influence both the occurrence and management of long‐term side effects in different regions. Expanding future research to include more diverse populations, as well as emerging treatment approaches, will be essential to improve the global relevance and applicability of this field.

## 5. Conclusion

Breast cancer treatment has made remarkable advancements, significantly improving survival rates. However, the long‐term side effects associated with these treatments present a substantial challenge to survivors’ overall well‐being. This systematic review highlights the persistent complications, including cardiovascular toxicity, cognitive impairment, fatigue, lymphedema, menopausal symptoms, and psychological distress. Addressing these challenges requires a comprehensive and multidisciplinary approach to survivorship care.

To mitigate long‐term effects, the following recommendations should be prioritized:

• Routine monitoring and early detection: Regular follow‐up screenings, including cardiovascular assessments, cognitive evaluations, and bone density scans, can aid in the early detection and management of treatment‐related complications.
• Personalized rehabilitation programs: Implementing structured rehabilitation programs, including physical therapy, cognitive training, and psychological counseling, can enhance recovery and improve quality of life.
• Lifestyle modifications: Encouraging survivors to engage in physical activity, maintain a balanced diet, and adopt stress management techniques such as yoga and mindfulness can help alleviate many long‐term effects.
• Improved treatment strategies: Research should focus on developing targeted therapies with fewer adverse effects and exploring cardioprotective agents and neuroprotective strategies to minimize toxicity.
• Psychosocial support services: Establishing accessible mental health services, peer support groups, and survivorship care plans can help breast cancer survivors cope with psychological distress and improve their overall well‐being.

In conclusion, while breast cancer treatments have significantly improved patient survival, their long‐term consequences must be addressed through proactive monitoring, multidisciplinary interventions, and patient‐centered care strategies. Future research and continued advancements in treatment approaches will be critical in improving the long‐term outcomes and quality of life for breast cancer survivors.

Breast cancer treatment has made remarkable advancements, leading to substantial improvements in survival rates. However, the long‐term side effects associated with these treatments continue to pose significant challenges to survivors’ overall well‐being. This systematic review highlights persistent complications, including cardiovascular toxicity, cognitive impairment, fatigue, lymphedema, menopausal symptoms, and psychological distress, many of which can last for years after treatment completion. Addressing these long‐term effects is therefore a critical component of comprehensive cancer care and survivorship planning.

To mitigate these adverse outcomes, survivorship care should prioritize routine monitoring and early detection through regular follow‐up assessments, including cardiovascular evaluation, cognitive screening, and bone density measurement. Personalized rehabilitation programs incorporating physical therapy, cognitive training, and psychological counseling may improve functional outcomes and quality of life. In addition, lifestyle modifications such as regular physical activity, a balanced diet, and stress management strategies can help alleviate several long‐term complications. Continued research is needed to develop treatment strategies with reduced toxicity, identify individuals at higher risk for late effects, and optimize multidisciplinary survivorship care models. Through proactive monitoring, individualized interventions, and integrated psychosocial support, long‐term outcomes and quality of life for breast cancer survivors can be meaningfully improved.

Future studies involving more diverse global populations and healthcare settings are needed to improve the generalizability of current evidence and strengthen survivorship care strategies for breast cancer survivors worldwide.

## Author Contributions

Ebrahim Babaee and Mahdi Soheyli contributed to the study conception and design. Ebrahim Babaee conducted the literature search, performed data extraction, and drafted the initial manuscript. Mahdi Soheyli performed the risk of bias assessment, assisted with data interpretation, and contributed to writing and revising the Results and Discussion sections. Nahid Nafissi supervised the project, resolved discrepancies during study selection, contributed to the interpretation of findings, and critically revised the manuscript for important intellectual content.

## Funding

No funding was received for this study.

## Disclosure

All authors reviewed and approved the final version of the manuscript.

## Ethics Statement

The research protocol received ethical approval from Iran University of Medical Science’s Institutional Review Board and Ethics Committee (IR.IUMS.REC.1404.547).

## Conflicts of Interest

The authors declare no conflicts of interest.

## Data Availability

As this study is a systematic review based exclusively on previously published literature, no new datasets were generated or analyzed during the current study. All data supporting the findings of this review are available within the cited articles and references included in the manuscript.
